# Scientific Items

**Published:** 1883-12-20

**Authors:** 


### SCIENTIFIC ITEMS.

**Gas Poisoning.**—Prof. M. Von Pettenkofer says it is a fact frequently proved that when a gas-main breaks in the street, people in the nearest houses are frequently taken sick and may even die. At all events death results from the carbonic oxide, of which there is about 10 per cent. in coal gas. It can always be detected in the blood of the sick or dead by Hoppe-Seyler's test. It is also a fact that such breaks are more dangerous in cold weather. The reason why more gas finds its way into the houses in winter than in summer is due only in part to the higher pressure on the gas during the long winter nights, as well as the frozen soil above has less penetrability, but far more to the important fact, which can be proved experimentally, that in winter the interior of the house acts like a chimney upon the air in the ground and cellars.

Max Graeber had already established the minimum limit for injurious quantities of carbonic oxide in the air, by a series of experiments upon animals, as 0·6 to 0·7 per thousand. There are decided symptoms of illness with 1·5 per thousand, which increases to 2 to 3·5 per thousand, without fatal results, even if such air is breathed for many hours. But when the quantity reaches 4 or 5 per thousand, fatal poisoning rapidly follows. Cramps set in with *episthotonus*, and the animals soon cease to breathe.

In one accident that occurred in Munich, where the room held 28 cubic meters (988 cubic feet) of air, 1·44 cubic meters (about 52 cubic feet) of coal gas sufficed, when mixed with the air, to reach 5 parts per million.

As a precaution against ground air contaminated with illuminating gas from entering houses, Von Pettenkofer recommends the police, the gas engineers, and private citizens to open all cellar windows as well as those on the ground floor of threatened houses, so as to prevent directly sucking in the ground air or render it harmless by dilution. Moreover, the smell of gas serves as a warning.—*Drug. Circular.*

**Metallized Wood.**—The following process, said to be of French origin, is given in Geyer's Stationer:

The wood is first immersed for three or four days, according to its permeability, in a caustic alkaline lye (lime and soda) at a temperature of from 75° to 90° C., (167° to 194° F.) From thence it passes immediately into a bath of hydrosulphate of calcium, to which is added, after 24 hours, a concentrated solution of sulphur in caustic potash. The duration of this bath is about 48 hours, and its temperature is from 35° to 50° C., (95° to 122° F.(. Finally the wood is immersed for 30 or 50 hours in a hot solution (35° to 50°) of acetate of lead. The process, as may be seen, is a long one, but the results are surprising. The wood thus prepared, after having undergone a proper drying at a moderate temperature, acquires under a burnish of hard wood a polished surface, and assumes a very brilliant metallic lustre. This lustre is still further increased if the surface of the wood be first rubbed with a piece of lead, tin or zinc, and be afterwards polished with a glass or porcelain burnisher. The wood thus assumes the appearance of a true metallic mirror, and is very solid and resistant. This should furnish the picture and photo frame-maders with a material for working up into a variety of new and attractive goods.—*Drug. Circular.*

**Climate and Character.**—An interesting summary of the opinions of a number of foreign savants concerning the influence of our climate in bringing out the peculiarities which distinguish the American character, is given by Mr. C. E. Young in a late issue of the Stanitarian. Dr. E. Reich, in noting the differences between the English and the Americans, although they are of the same race, is quoted as asserting that the differences in question are ascribable to the contrasts of the climates of the two countries. The American atmosphere, he says, "is much too dry for the Anglo-Saxon race, and in point of heat too excessive; from this results the exaggerated nervous activity, the excesses of the national character, and the mad chase after the material things of the world."

Dr. Pettendofer ascribes the nervous temperament characteristic of the American people, to the dryness of our climate. He has investigated the comparative loss of heat suffered by a person breathing moist and dry air, and finds that more heat is lost in the latter case than in the former, and that more is created. In consequence of this, the circulation is quicker and more intense, life is more energetic, and there is no opportunity for the development of an excessive amount of flesh or fat.

Essentially the same conclusions are reached by Dr. De Pietro Santa. Dr. Ludwig Buchner, a well-known German author, says that he has noticed among Americans a tendency towards the typical characteristics of the Indians—in face and form, gestures and movements. Dr. Carl Reclam compares the air of this country with that existing in elevated regions, its lightness and dryness producing substantially the same effects as the air of heights upon the physical and mental peculiarities of Americans.

Mr. Young finally gives his own conclusions in the following language: "The dry air with us produces nervous, energetic, large-jointed skeletons, which have little or nothing in common with the stout, fresh, rosy, phlegmatic inhabitants of the mother country. Not only is the physical resemblance lost in the second generation, but the mental also, and ideas especially Britannic give way to ideas peculiarly American."—*Ibid.*

**Protective Coating for Glass.**—Mr. Schael proposes for the protection of retorts and other apparatus of glass exposed to high temperatures, a mixture of kieselguhr (infusorial earth) and of soluble sodium silicate. This mass is applied as a soft paste so as to form a coating of 5 to 10 m.m. in thickness. The articles are then dried slowly.—*Exchange.*

